# 

**Published:** 1905-11

**Authors:** 


VOL. XXI. NOVEMBER, 1905. No. 5.

### TEXAS Medical Journal.

SUBSCRIPTION, $1.00 A YEAR, IN ADVANCE.

SINGLE COPIES, 10 CENTS.

[illegible] SURGEON GENERAL'S OFFICE
NOV. 18 1905

LIBRARY
INDEXED
S. G. O.

PUBLISHED AT AUSTIN, TEXAS, BY

F. E. DANIEL, M. D.,

PROPRIETOR.

PRINTED BY THE VON BOECKMANN-JONES CO.

Entered at the Postoffice at Austin, Texas, as Second-class Mail Matter.

